# Clinical and Imaging Markers of Cardiac Function and Brain Health: A Meta-Analysis of Community-Based Studies

**DOI:** 10.1212/WNL.0000000000213421

**Published:** 2025-03-26

**Authors:** Amber Yaqub, Joshua C. Bis, Stefan Frenzel, Marisa Koini, Djass Mbangdadji, Gina M. Peloso, Rajesh Talluri, Alvaro Alonso, Martin Bahls, Robin Bülow, Marcus Dörr, Stephan Felix, Alison Fohner, Nele Friedrich, Edith Hofer, Maryam Kavousi, Lenore J. Launer, Tran Le, Will Longstreth, Thomas H. Mosley, Meike W. Vernooij, Henry Völzke, Katharina Wittfeld, Alexa S. Beiser, Hans J. Grabe, Vilmundur Gudnason, Mohammad Arfan Ikram, Bruce M. Psaty, Reinhold Schmidt, Jeannette Simino, Sudha Seshadri, Frank J. Wolters, as Cross-Cohort Collaboration

**Affiliations:** 1 Department of Epidemiology, Erasmus MC, Rotterdam, the Netherlands;; 2 Cardiovascular Health Research Unit, Department of Medicine, University of Washington, Seattle;; 3 Department of Psychiatry and Psychotherapy, University Medicine Greifswald, Germany;; 4 Division of Neurogeriatrics, Department of Neurology, Medical University of Graz, Austria;; 5 Laboratory of Epidemiology and Population Sciences, Intramural Research Program, National Institute on Aging, Baltimore, MD;; 6 Department of Neurology, The University of Texas Health Science Center at San Antonio;; 7 Glenn Biggs Institute for Alzheimer and Neurodegenerative Diseases, The University of Texas Health Science Center at San Antonio;; 8 Department of Data Science, John D. Bower School of Population Health, University of Mississippi Medical Center, Jackson;; 9 Department of Epidemiology, Rollins School of Public Health, Emory University, Atlanta, GA;; 10 Department of Internal Medicine B, University Medicine Greifswald, Germany;; 11 Institute of Diagnostic Radiology and Neuroradiology, University Medicine Greifswald, Germany;; 12 Department of Epidemiology, University of Washington, Seattle;; 13 Institute of Clinical Chemistry and Laboratory Medicine, University Medicine Greifswald, Germany;; 14 German Centre for Cardiovascular Research (DZHK), Partner Site Greifswald, Greifswald, Germany;; 15 Department of Neurology, University of Washington, Seattle;; 16 Gertrude C. Ford Memory Impairment and Neurodegenerative Dementia (MIND) Center, University of Mississippi Medical Center, Jackson;; 17 Department of Medicine and Neurology, University of Mississippi Medical Center, Jackson;; 18 Department of Radiology and Nuclear Medicine, Erasmus University Medical Center, Rotterdam, the Netherlands;; 19 Institute for Community Medicine, University Medicine Greifswald, Germany;; 20 Department of Biostatistics, Boston University School of Public Health, MA;; 21 Department of Neurology, Boston School of Medicine, MA;; 22 Icelandic Heart Association, Kopavogur, Iceland;; 23 Faculty of Medicine, University of Iceland, Reykjavik;; 24 Department of Health Systems and Population Health, University of Washington, Seattle; and; 25 Framingham Heart Study, Framingham, MA.

## Abstract

**Background and Objectives:**

Cardiac dysfunction and heart failure are linked to cognitive impairment, but the underlying brain pathology remains undetermined. We investigated associations between cardiac function (measured by echocardiography or cardiac MRI), clinical heart failure, and structural markers on brain MRI, including volumes of gray and white matter (WM), the hippocampus, and white matter hyperintensities (WMHs).

**Methods:**

We leverage data from 7 prospective, community-based cohorts across Europe and the United States, all part of the Cross-Cohort Collaboration. The included cohorts were the Age, Gene/Environment Susceptibility-Reykjavik Study, Atherosclerosis Risk in Communities study, Austrian Stroke Prevention Study, Cardiovascular Health Study, Framingham Heart Study, Rotterdam Study, and Study of Health in Pomerania (SHIP-START and SHIP-TREND). Each cohort performed cross-sectional multivariable linear regression analyses, after which estimates were pooled through random-effects meta-analysis. Heterogeneity was assessed by the *I*^2^ index (%).

**Results:**

Among 10,889 participants (mean age: 66.8 years, range 52.0–76.0; 56.7% women), markers of systolic dysfunction were consistently associated with smaller total brain volume (TBV) (e.g., adjusted standardized mean difference for moderate to severe dysfunction −0.19, 95% CI −0.31 to −0.07, *I*^2^ = 20%). Impaired relaxation and restrictive diastolic dysfunction were also associated with smaller TBV (e.g., for impaired relaxation −0.08, 95% CI −0.15 to −0.01, *I*^2^ = 32%) and hippocampal volume (−0.18, 95% CI −0.33 to −0.03, *I*^2^ = 0%), with similar results for the E/A-ratio. Systolic and diastolic dysfunction was not consistently associated with volume of WMHs. Among 5 cohorts with available data, 302 (3.4%) participants had clinical heart failure, which was associated with smaller brain volumes, particularly in the hippocampus (−0.13, 95% CI −0.23 to −0.02, *I*^2^ = 1%).

**Discussion:**

In this large study among community-dwelling adults, subclinical cardiac dysfunction was associated with brain imaging markers of neurodegeneration. These findings encourage longitudinal investigations on the effect of maintaining cardiac function on brain health.

## Introduction

Heart failure is increasingly recognized as an important risk factor for neuronal injury,^1^ cognitive impairment,^2,3^ and dementia.^4,5^ Depending on the applied criteria, 25%–70% of patients with heart failure experience cognitive impairment, causing poorer therapeutic compliance and higher risk of functional decline.^2^ Early identification and treatment of cardiac dysfunction is crucial for maintaining cognitive abilities later in life, but the development of preventive measures is hindered by a limited understanding of the pathophysiologic mechanisms that link heart failure to dementia.

Mechanisms that connect cardiac dysfunction to dementia include chronic hypoxia, release of proinflammatory factors, recurrent thromboembolic events, effects of natriuretic peptides, and other shared pathologic substrates along the heart-brain axis.^2,6^ Experimental studies in mice have demonstrated that chronic cerebral hypoperfusion results in white matter (WM) changes, hippocampal atrophy, neuronal death, and memory impairment.^7^ Supporting this, a 2-sample Mendelian randomization study showed that heart conditions may be causally related to structural brain abnormalities.^8^ Importantly, cardiac dysfunction may contribute to neuronal and cerebrovascular injury already from midlife,^9,10^ when cognitive deficits are generally absent, but neuroimaging may show subtle alterations in brain structure that increase the susceptibility for dementia. Indeed, some studies have reported smaller brain volumes^11,12^ and a greater burden of cerebral small vessel disease^13-15^ in individuals with clinical or subclinical cardiac dysfunction, while others could not confirm such associations.^9,10,16,17^ Methodologic heterogeneity in the assessment of cardiac function (e.g., cardiac index, cardiac output, and ejection fraction) has further hampered the interpretation of the observed associations. Moreover, the available evidence predominantly derives from small scale clinical samples that may represent individuals in advanced stages of disease,^14,18,19^ and whether and how these findings apply to the general population remains unclear. Another prevailing issue pertains to the assessment of diastolic dysfunction, or heart failure with preserved ejection fraction (HFpEF), for which the recently updated criteria only seem to identify the most severe cases in the population.^20^ We have previously shown that even mild diastolic dysfunction in community-dwelling adults was associated with increased prevalence of clinically defined stroke, covert infarcts on brain MRI, and dementia, while worse systolic function was only associated with clinical stroke.^21^ By harmonizing continuous measures of cardiac function and high-resolution brain MRI across studies, we may be able to identify pathophysiologic correlates of cardiac dysfunction with more precision, including subtle neurodegenerative and cerebrovascular changes that could contribute to cognitive decline, but not (yet) to the clinical stage of dementia.

In this Cross-Cohort Collaboration (CCC), we aggregated results from 7 large community-based studies across Europe and the United States to determine associations between cardiac function, structural imaging markers of neurodegeneration, and cerebral small vessel disease on brain MRI.

## Methods

### Study Population: The CCC Consortium

The neurology working group of CCC comprises multiple population-based cohorts including the Age, Gene/Environment Susceptibility (AGES)-Reykjavik Study, the Atherosclerosis Risk in Communities (ARIC) study, the Austrian Stroke Prevention Study (ASPS), the Cardiovascular Health Study (CHS), the Framingham Heart Study (FHS), the Rotterdam Study (RS), the Study of Health in Pomerania (SHIP-START and SHIP-TREND), and the Three-City Study. Seven of 8 cohorts could participate in this study, including 10,889 participants who underwent echocardiography before volumetric brain MRI, and 2,745 participants with data on cardiac MRI before brain MRI. Individuals with prevalent dementia, poor quality echocardiography, brain abnormalities that substantially affected segmentations, and those with atrial fibrillation at time of echocardiography or cardiac MRI were excluded. This is because irregular heart rhythms in atrial fibrillation complicate a reliable assessment of cardiac imaging markers. A detailed description of each included cohort can be found in eMethods 1.

### Standard Protocol Approvals, Registrations, and Patient Consents

Ethical approval was obtained from the institutional review boards of all studies included in the CCC, and all study participants provided written informed consent. Additional details on ethics, funding, and acknowledgement per cohort are noted in eAppendix 1.

### Assessment of Cardiac Function

Echocardiographic evaluation of cardiac function was conducted in 2 dimensions, in the M-mode, pulsed wave Doppler and color Doppler using the parasternal short axis, parasternal long axis, apical 2-, 4-, or 5-chamber view, and subcostal view. Wall motion abnormalities were taken into account during assessment of echocardiographic function. The 2 measures used to assess systolic function were fractional shortening (FS) and left ventricular ejection fraction. FS (in %) was determined by the difference of the left ventricular end-diastolic diameter (LVEDD) and the left ventricular end-systolic diameter/LVEDD × 100%. Left ventricular ejection fraction (LVEF, in %) was quantified by the Simpson biplane method, and if unavailable, estimated by the Teichholz formula: 7 × D^3^/(2.4 + D), where D represents the diameter of the left ventricle (LV) in millimeters. Subsequently, systolic function was categorized normal (LVEF ≥50%), mild systolic dysfunction (LVEF 40%–49%), or moderate to severe systolic dysfunction (LVEF <40%), based on previously recommended screening cutoffs.^22^ The measures for determining diastolic function included the mitral inflow deceleration time (in milliseconds) and the ratio between the transmitral flow velocity during early (E-wave) and late (A-wave) diastole (i.e., the EA-ratio). Accordingly, diastolic function was categorized normal (EA-ratio 0.75–1.50 and deceleration time 150–280 milliseconds), impaired relaxation (EA-ratio <0.75 and deceleration time >280 milliseconds), restrictive pattern (EA-ratio >1.50 and deceleration time <150 milliseconds), or indeterminate (any abnormal combination that did not fulfil criteria for impaired relaxation or restrictive pattern dysfunction).^23^ Cardiac MRI was performed using a 1.5T scanner. For extended details on cardiac imaging per study cohort, we refer to eMethods 2.

### Assessment of Clinical Heart Failure

Definition of clinical heart failure was based on criteria proposed by the European Society of Cardiology.^24^ Cases of heart failure were verified by medical records in all cohorts. Case ascertainment of clinical heart failure was based on medical history, typical signs and symptoms of heart failure (i.e., shortness of breath, edema, paroxysmal nocturnal dyspnea, and orthopnea), signs at physical examination (such as bilateral ankle edema or jugular vein distension), that had to be supported by some objective evidence of cardiac dysfunction on imaging (chest X-ray, echocardiography, and laboratory assessment). For descriptions of case ascertainment of clinical heart failure per cohort, we refer to eMethods 3.

### Neuroimaging Protocols

Neuroimaging was performed using 1.5T scanners in all cohorts, except for ARIC that used 3T scanners. In compliance with standard protocols, volumes of total intracranial volume, total brain, gray matter (GM), WM, hippocampus, and white matter hyperintensities (WMHs) were determined in mm^3^ using T1-weighted and fluid-attenuated inversion recovery MRI data. Segmentations were performed using in-house pipelines or publicly available software, such as FreeSurfer or the FMRIB Software library.^25^ Additional specifics of the neuroimaging protocols are noted in our earlier publications, and a summary is provided in eMethods 4.

### Covariable Assessment

Covariables were harmonized as much as possible across cohorts within the CCC, as described in one of our earlier publications,^26^ and measured at the time of cardiac assessment. Information on smoking habits was obtained during interviews, and participants were categorized never or ever smokers. Data on medication use (blood pressure–lowering medication or lipid-lowering medication) also derived from interview data. Ancestry was self-reported in some cohorts, and confirmed by genetic analysis in others (eMethods 5), after which participants were categorized White or non-White. Height (in meters), weight (in kilograms), and blood pressure (in mm Hg) were measured when participants visited the research center. The body mass index was calculated by dividing weight (in kilograms) by height (in meters) squared. Blood samples that were obtained during center visits provided information on total cholesterol, high-density lipoprotein (HDL) cholesterol, glucose levels in mmol/L, and *APOE*-ε4 carrier status. Medical history of coronary heart disease (CHD) and atrial fibrillation was self-reported during interviews, and verified with medical records.

### Statistical Analysis

Missing covariables were imputed with the mean of fivefold multiple imputation if missings exceeded 5%, which applied to the RS and the (AGES)-Reykjavik Study. If missings led to exclusion of <5% of the data, we performed complete case analysis.

First, we applied a square root transformation to FS, and a natural log transformation to deceleration time and volume of WMHs, to address the skewness and deviations from normal distributions of the data. Afterward, both cardiac function measures and neuroimaging markers were standardized to facilitate comparison. Given that the EA-ratio can increase, after an initial decrease, to turn pseudonormal with progressively worse diastolic function,^23^ we also included a quadratic term (EA-ratio^2^) during analysis.

We then determined associations between cardiac function parameters (FS, LVEF, EA-ratio, and deceleration time) and all structural neuroimaging markers using linear regression models to present mean differences with corresponding 95% CIs within each cohort. Model I was adjusted for age-specific, age^2^-specific, sex-specific, and cohort-specific covariables (e.g., study site, cohort, and scanner). Model II was additionally adjusted for *APOE*-ε4 carriership, smoking, body mass index, ancestry, systolic blood pressure, diastolic blood pressure, use of blood pressure–lowering medication, total cholesterol, HDL-cholesterol, use of lipid-lowering medication, diabetes, and history of CHD. Given the potential effect of irregular heart rhythms on brain structure, Model III was additionally adjusted for a history of atrial fibrillation. In all neuroimaging models, adjustments were made for total intracranial volume and, if applicable, the time interval between the assessment of cardiac function and brain MRI. For models specifically related to WMHs, additional adjustments were made for normal appearing WM volume. Above-mentioned analyses were repeated for model II after (1) excluding participants with clinical heart failure and (2) excluding participants with prevalent infarcts on brain MRI. In addition, we performed age-stratified analyses across all cohorts for model II with the following age categories: <40, 40–49, 50–59, 60–69, 70–79, and ≥80 years. Finally, within the RS only, we determined sex-specific associations for men and women separately.

Effect estimates (adjusted standardized mean differences) from each study were pooled using a random-effects meta-analysis using the inverse variance method and the DerSimonian-Laird estimator, after which statistical heterogeneity was assessed by the *I*^2^ and corresponding *p* value for heterogeneity. The nominal significance threshold was an α of 0.05 (*p* ≤ 0.05). Multiple testing correction was not applied because methods such as Bonferroni can be overly conservative for correlated predictors (e.g., cardiac measures), introducing the possibility of a type II error.^27^ Meta-analysis was performed using R version 3.6.1 (packages tidyr, dplyr, lubridate, foreign, and meta).

### Data Availability

Reasonable requests for access to data will be considered. Such requests can be directed toward the management team of the RS (secretariat.epi@erasmusmc.nl), which adheres to a protocol for evaluating such requests. Owing to privacy regulations and informed consent provided by participants, the data cannot be uploaded to a public repository.

## Results

Characteristics of each cohort are listed in Table 1. A total of 10,889 participants were included with a median age ranging from 52.0 years in SHIP-TREND to 76.0 years in ARIC. Across all cohorts, women outnumbered men. ARIC and CHS had the most ethnically diverse populations (28.6% and 13.2% were non-White, respectively). Although most participants had a history of smoking, participants from FHS generally comprised of never smokers (92.9%). Other notable differences were in use of blood pressure–lowering medication (ranging from 30.3% in SHIP-START to 75.7% in ARIC) and use of lipid-lowering medication (9.1% in CHS to 54.9% in ARIC), despite similar blood pressure and cholesterol levels across cohorts. The results presented in this section are based on model II because additional adjustment for atrial fibrillation had little to no effect on the observed mean differences.

**Table 1:** Baseline Characteristics of the Study Population per Cohort

	AGES (n = 752)	ARIC (n = 1,864)	ASPS (n = 37)	CHS (n = 770)	FHS (n = 1,444)	RS (n = 4,272)	SHIP-START (n = 1,054)	SHIP-TREND (n = 2,070)
Sex, female, n (%)	431 (57.3)	1,119 (60.0)	23 (62.2)	459 (59.6)	788 (54.6)	2,373 (55.5)	554 (52.6)	1,070 (51.7)
Age at echocardiography, y, median (IQR)	75.0 (71.0–80.0)	76.0 (72.0–80.0)	72.3 (67.5–76.9)	75.0 (72.0–78.0)	65.0 (59.0–72.0)	61.9 (56.3–68.7)	55.0 (44.0–65.0)	52.0 (41.0–62.0)
Age at echocardiography, y, mean (SD)	75.4 (5.4)	76.3 (5.3)	72.8 (5.6)	75.5 (4.2)	65.3 (8.8)	62.7 (8.8)	55.2 (12.6)	51.1 (14.0)
Ethnicity, n (%)								
Non-White	0 (0.0)	534 (28.6)	0 (0.0)	102 (13.2)	0 (0.0)	203 (4.9)	0 (0)	0 (0)
White	752 (100.0)	1,330 (71.4)	37 (100.0)	668 (86.8)	1,444 (100.0)	3,943 (95.1)	1,054 (100)	2,070 (100)
*APOE* ε4 carriership, n (%)								
No carriers	562 (74.7)	1,291 (71.7)	33 (89.2)	579 (75.2)	1,086 (77.4)	2,890 (72.1)	806 (76.5)	1,451 (70.1)
Carriers	190 (25.3)	510 (28.3)	4 (10.8)	191 (24.8)	317 (22.6)	1,119 (27.9)	218 (20.7)	466 (22.5)
Smoking, n (%)								
Never	328 (43.6)	765 (43.1)	20 (54.1)	359 (46.6)	1,342 (92.9)	1,274 (30.2)	398 (37.8)	821 (39.7)
Ever	424 (56.4)	1,011 (56.9)	17 (45.9)	411 (53.4)	102 (7.1)	2,287 (54.2)	656 (62.2)	1,249 (60.3)
Body mass index, kg/m^2^	27.0 (4.4)	28.5 (5.7)	27.6 (4.0)	26.7 (4.4)	27.9 (5.0)	27.3 (4.0)	27.6 (4.4)	27.6 (4.5)
Systolic blood pressure, mm Hg	142.2 (19.5)	130.9 (18.3)	141.9 (17.3)	130.8 (19.4)	127.9 (17.0)	138.9 (20.1)	131.6 (18.2)	126.5 (17.3)
Diastolic blood pressure, mm Hg	74.2 (9.1)	66.1 (10.6)	85.9 (10.8)	68.8 (10.8)	73.8 (9.5)	80.9 (10.6)	80.5 (10.0)	77.2 (9.8)
Use of blood pressure–lowering medication, n (%)	455 (60.5)	1,411 (75.7)	12 (32.4)	368 (47.8)	643 (44.6)	1,310 (30.9)	319 (30.3)	688 (33.2)
Total cholesterol, mmol/L	5.6 (1.1)	4.7 (1.1)	0.4 (0.1)	5.2 (1.0)	4.86 (1.0)	5.6 (1.0)	5.5 (1.1)	5.5 (1.1)
HDL cholesterol, mmol/L	1.6 (0.4)	1.4 (0.4)	0.1 (0.03)	1.4 (0.4)	1.5 (0.5)	1.5 (0.4)	1.5 (0.4)	1.5 (0.4)
Use of lipid-lowering medication, n (%)	178 (23.7)	1,020 (54.9)	3 (8.1)	70 (9.1)	590 (40.9)	922 (21.8)	164 (15.6)	201 (9.7)
Type 2 diabetes, n (%)	99 (13.2)	606 (32.5)	1 (2.7)	73 (9.5)	154 (10.8)	408 (9.7)	87 (8.3)	175 (8.5)
History of CHD, n (%)	160 (21.3)	180 (9.8)	0 (0.0)	148 (19.2)	116 (8.0)	219 (5.2)	19 (1.8)	24 (1.2)
History of atrial fibrillation, n (%)	27 (3.6)	118 (6.4)	0 (0.0)	26 (3.4)	53 (3.7)	77 (1.8)	32 (3.0)	74 (3.6)
Cardiac function parameters								
Fractional shortening value, n (%)	33.5 (8.6)	40.8 (7.7)	39.0 (8.5)	42.2 (8.3)	37.8 (5.4)	41.0 (5.7)	40.7 (7.9)	41.7 (8.2)
Left ventricular ejection fraction value, n (%)	62.7 (6.6)	65.8 (6.3)	73.9 (8.1)	N/A	67.4 (6.9)	77.3 (6.9)	70.6 (9.5)	71.6 (9.6)
Left ventricular ejection fraction categories, n (%)								
Normal (LVEF ≥50%)	630 (83.8)	1,762 (98.1)	N/A	N/A	1,297 (98.6)	4,246 (99.4)	816 (77.4)	1,679 (81.1)
Mild systolic dysfunction (LVEF 40%–49%)	4 (0.5)	25 (1.4)	N/A	N/A	15 (1.1)	15 (0.4)	19 (1.8)	27 (1.3)
Moderate to severe dysfunction (LVEF <40%)	11 (1.5)	9 (0.5)	N/A	N/A	4 (0.3)	11 (0.3)	7 (0.7)	12 (0.6)
Mitral valve inflow deceleration time, msec	263.8 (62.0)	205.0 (45.3)	N/A	N/A	N/A	200.7 (38.4)	189.1 (42.6)	178.4 (36.4)
E/A ratio (mitral valve velocity peak E/peak A)	0.9 (0.3)	0.9 (0.3)	N/A	1.0 (0.4)	0.9 (0.3)	1.0 (0.3)	1.1 (0.3)	1.2 (0.4)
Diastolic function categories, n (%)								
Normal	331 (44.0)	921 (51.4)	N/A	N/A	N/A	3,149 (73.7)	623 (59.1)	1,191 (57.5)
Impaired	90 (12.0)	61 (3.4)	N/A	N/A	N/A	78 (1.8)	15 (1.4)	9 (0.4)
Restrictive	2 (0.3)	8 (0.4)	N/A	N/A	N/A	22 (0.5)	0 (0.0)	0 (0.0)
Indeterminate	329 (43.7)	802 (44.8)	N/A	N/A	N/A	1,023 (23.9)	388 (36.8)	794 (38.4)
Heart failure								
Clinical heart failure present, n (%)	25 (3.3)	187 (10.0)	N/A	30 (3.9)	15 (1.0)	45 (1.1)	N/A	N/A

Abbreviations: AGES = Age, Gene/Environment Susceptibility Study; ARIC = Atherosclerosis Risk in Communities Study; ASPS = Austrian Stroke Prevention Study; CHD = coronary heart disease; CHS = Cardiovascular Health Study; FHS = Framingham Heart Study; HDL = high-density lipoprotein; IQR = interquartile range; LVEF = left ventricular ejection fraction; n = number of participants; RS = Rotterdam Study; SHIP = Study of Health in Pomerania.

Unless specified otherwise, mean values and SD are displayed for continuous measures and frequencies (%) are presented for categorical values.

### Systolic Function and Brain Volumes

**Figure 1:**
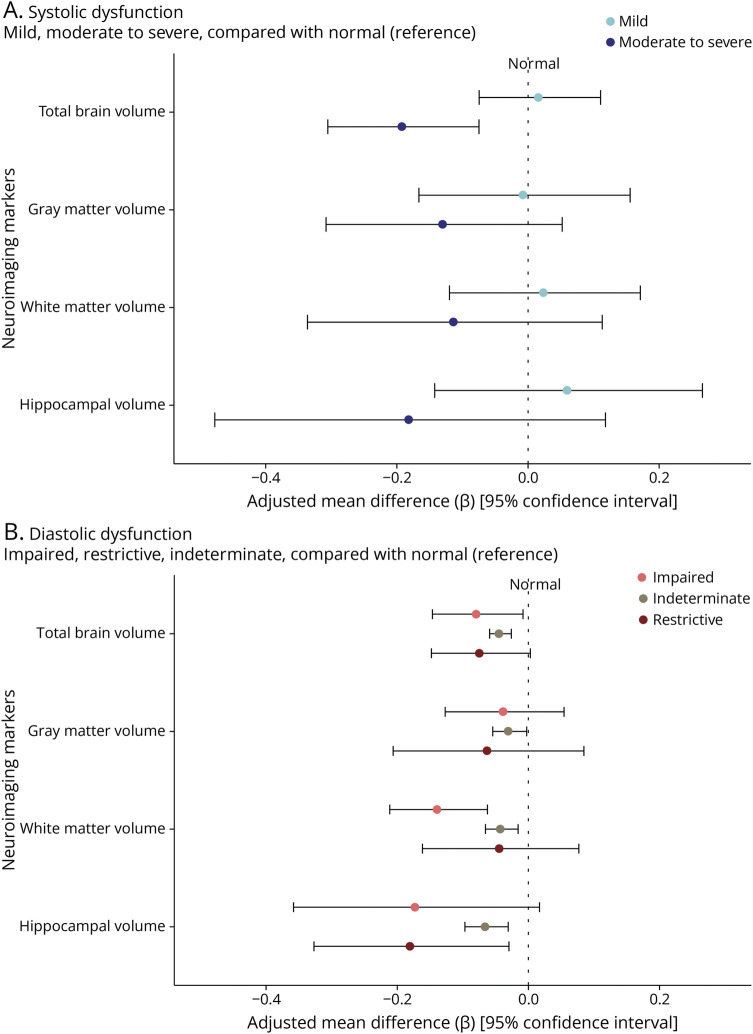
Systolic (A) or Diastolic Function (B) and Brain Volumes Associations between systolic (A) and diastolic function (B) on echocardiography and brain volumes on MRI are depicted as adjusted mean differences, with 95% CIs. Note that neuroimaging markers were standardized (displayed per 1 SD) to facilitate comparison. The normal category is used as reference. Extended details of each plot can be found in eTable 1. Cohorts included for the meta-analysis of systolic dysfunction (n = 10,302): AGES, ARIC, FHS, RS, SHIP-START, and SHIP-TREND. Cohorts included for the meta-analysis of diastolic dysfunction (n = 9,556): AGES, ARIC, RS, SHIP-START, and SHIP-TREND. AGES = Age, Gene/Environment Susceptibility Study; ARIC = Atherosclerosis Risk in Communities Study; CHS = Cardiovascular Health Study; FHS = Framingham Heart Study; RS = Rotterdam Study; SHIP = Study of Health in Pomerania.

Figure 1A illustrates the associations between systolic function, indicated by left ventricular ejection fraction, and brain volumes. Notably, moderate to severe systolic dysfunction, compared with normal systolic function, was associated with a smaller total brain volume (TBV) (adjusted standardized mean difference −0.19; 95% CI −0.31 to −0.07, *I*^2^ = 20.3%, *p*_Heterogeneity_ = 0.28). These results were comparable for GM and WM, and somewhat more pronounced in the hippocampus, although subregional analyses were not statistically significant (see eTable 1 for details). In accordance, higher left ventricular ejection fraction (eFigure 1) and FS (eFigure 2), which are both continuous measures of systolic function, were generally associated with larger brain volumes. In cohorts that had access to cardiac MRI (FHS, SHIP-START, and SHIP-TREND), associations between LVEF and hippocampal volumes (HVs) were consistent with echocardiography defined LVEF, indicating no heterogeneity (0.04, 95% CI 0.01–0.06, *I*^2^ = 0%, *p*_Heterogeneity_ = 0.69; eFigure 3).

### Diastolic Function and Brain Volumes

**Figure 2:**
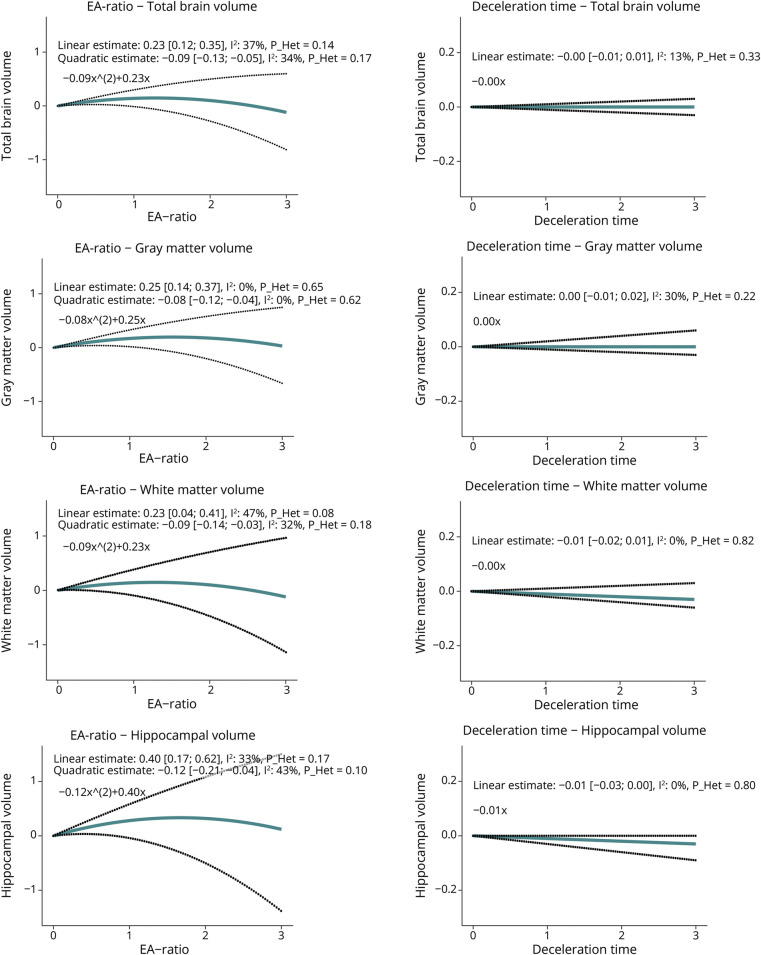
Associations Between EA-Ratio or Deceleration Time and Brain Volumes Associations between EA-ratio or deceleration time on echocardiography and brain volumes on MRI were obtained using a random-effects meta-analysis (DerSimonian and Laird method). Estimates are displayed as adjusted mean differences, with 95% CIs. Note that all echocardiographic and neuroimaging markers were standardized (displayed per 1 SD) to facilitate comparison. Given that the EA-ratio is known to be nonlinear (U-shaped) in progressively worse diastolic function, a quadratic term (EA-ratio^2^) was included in the model. The figure displays the formula for the pooled adjusted mean differences, which constitutes of a quadratic estimate and/or a linear estimate. Model 2 is displayed, which is adjusted for age-specific, age^2^-specific, sex-specific, cohort-specific covariates, *APOE*-ε4 carriership, smoking, body mass index, ethnicity, systolic blood pressure, diastolic blood pressure, use of blood pressure–lowering medication, total cholesterol, high-density lipoprotein-cholesterol, use of lipid-lowering medication, diabetes, and history of coronary heart disease. Details can be found in eFigure 4.

Impaired relaxation during diastole, compared with normal diastole, was associated with smaller TBV (−0.08, 95% CI −0.15 to −0.01, *I*^2^ = 32.3%, *p*_Heterogeneity_ = 0.21), more profound for WM than for GM volume. HVs were not statistically different (Figure 1B). Similar patterns were observed for restrictive diastolic function, which was associated with a significantly smaller HV (−0.18, 95% CI −0.33 to −0.03, *I*^2^ = 0%, *p*_Heterogeneity_ = 0.59; Figure 1B, eTable 1). Abnormal diastolic function that could not otherwise be classified (“indeterminate”) was also consistently associated with lower volumes of total brain, WM, and the hippocampus. Among separate components of diastolic function (deceleration time and EA-ratio), associations with brain volumes seemed to be driven by the EA-ratio rather than deceleration time. We illustrate this for all brain volumes in Figure 2, and refer to eFigure 4 for additional details.

### Clinical Heart Failure and Brain Volumes

**Figure 3:**
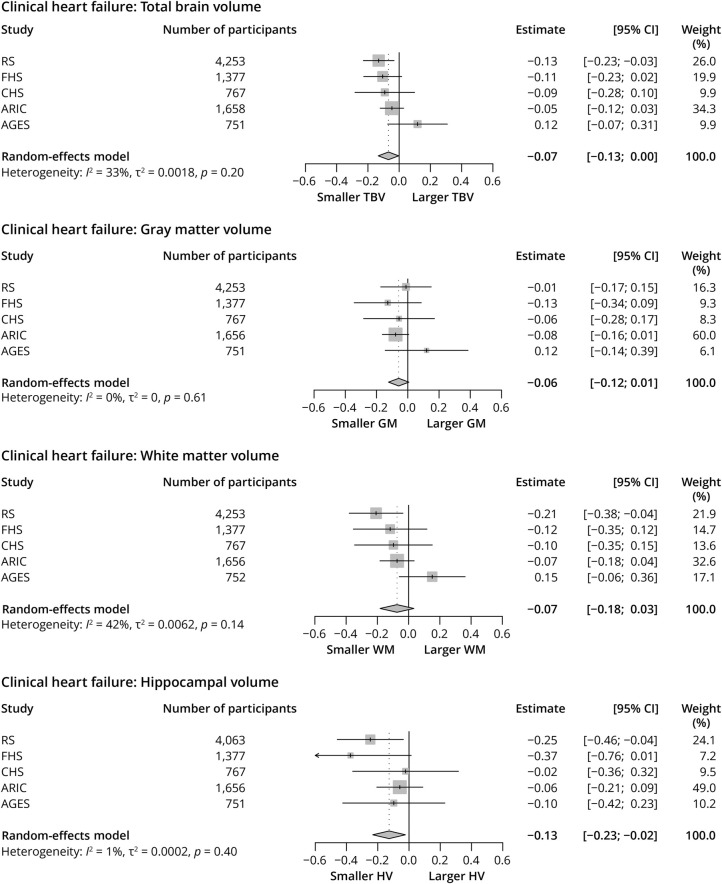
Associations Between Clinical Heart Failure and Brain Volumes Associations between clinical heart failure and brain volumes on MRI were pooled using a random-effects meta-analysis (DerSimonian and Laird method). Estimates are displayed as adjusted mean differences, with 95% CIs. Absence of clinical heart failure was used as reference. All neuroimaging markers were standardized (displayed per 1 SD) to facilitate comparison. Model 2 is displayed, which is adjusted for age-specific, age^2^-specific, sex-specific, cohort-specific covariates, *APOE*-ε4 carriership, smoking, body mass index, ethnicity, systolic blood pressure, diastolic blood pressure, use of blood pressure–lowering medication, total cholesterol, high-density lipoprotein-cholesterol, use of lipid-lowering medication, diabetes, and history of coronary heart disease. AGES = Age, Gene/Environment Susceptibility Study; ARIC = Atherosclerosis Risk in Communities Study; CHS = Cardiovascular Health Study; FHS = Framingham Heart Study; GM = gray matter; HV = hippocampal volume; RS = Rotterdam Study; TBV = total brain volume; WM = white matter.

The prevalence of clinical heart failure in community-dwelling adults, across the 5 cohorts, was 3.4% (n = 302 out of N = 8,806), with the largest share of participants with clinical heart failure originating from ARIC (10.0%). In accordance with observations for systolic and diastolic function, clinical heart failure was consistently and significantly associated with smaller volumes of total brain (−0.07, 95% CI −0.13 to −0.00, *I*^2^ = 33%, *p*_Heterogeneity_ = 0.20) and the hippocampus (−0.13, 95% CI −0.23 to −0.02, *I*^2^ = 1%, *p*_Heterogeneity_ = 0.40), while comparable estimates were observed for GM and WM (Figure 3).

### Cardiac Function and Markers of Cerebral Small Vessel Disease

**Figure 4:**
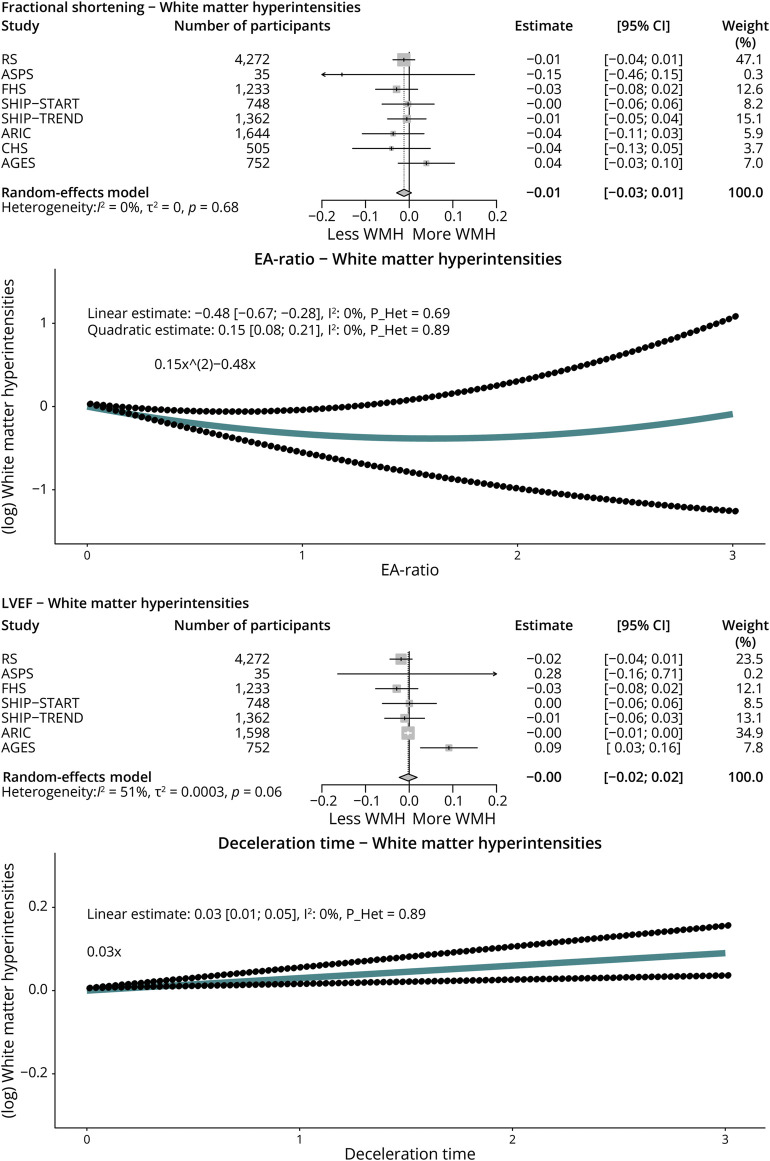
Cardiac Function on Echocardiography and Cerebral Small Vessel Disease Associations between echocardiographic measures and white matter hyperintensities (markers of cerebral small vessel disease) on MRI were obtained using a random-effects meta-analysis (DerSimonian and Laird method). Estimates are displayed as adjusted mean differences, with 95% CIs. All echocardiographic and neuroimaging markers were standardized (displayed per 1 SD) to facilitate comparison. Given the nonlinear (U-shaped) association of the EA-ratio in worsening diastolic function, we included a quadratic term (EA-ratio^2^) in the model. Pooled adjusted mean differences for both the EA-ratio and the deceleration time are shown. Model 2 is displayed, which is adjusted for age-specific, age^2^-specific, sex-specific, cohort-specific covariates, *APOE*-ε4 carriership, smoking, body mass index, ethnicity, systolic blood pressure, diastolic blood pressure, use of blood pressure–lowering medication, total cholesterol, high-density lipoprotein-cholesterol, use of lipid-lowering medication, diabetes, and history of coronary heart disease. Categorical associations (systolic and diastolic function) are displayed in eFigure 5A and B. AGES = Age, Gene/Environment Susceptibility Study; ARIC = Atherosclerosis Risk in Communities Study; CHS = Cardiovascular Health Study; FHS = Framingham Heart Study; RS = Rotterdam Study; SHIP = Study of Health in Pomerania; WMH = white matter hyperintensity.

Increasingly worse systolic function (from mild to moderate or severe) was not significantly associated with more WMHs (eFigure 5A). Volume of WMHs was higher with a higher deceleration time (0.03, 95% CI 0.01–0.05, *I*^2^ = 0%, *p*_Heterogeneity_ = 0.88) (Figure 4) but did not differ by categories of diastolic dysfunction (eFigure 5B). Volume of WMHs was somewhat higher in participants with clinical heart failure, but this association was not statistically significant (eFigure 5C).

### Sensitivity and Stratified Analyses

**Figure 5:**
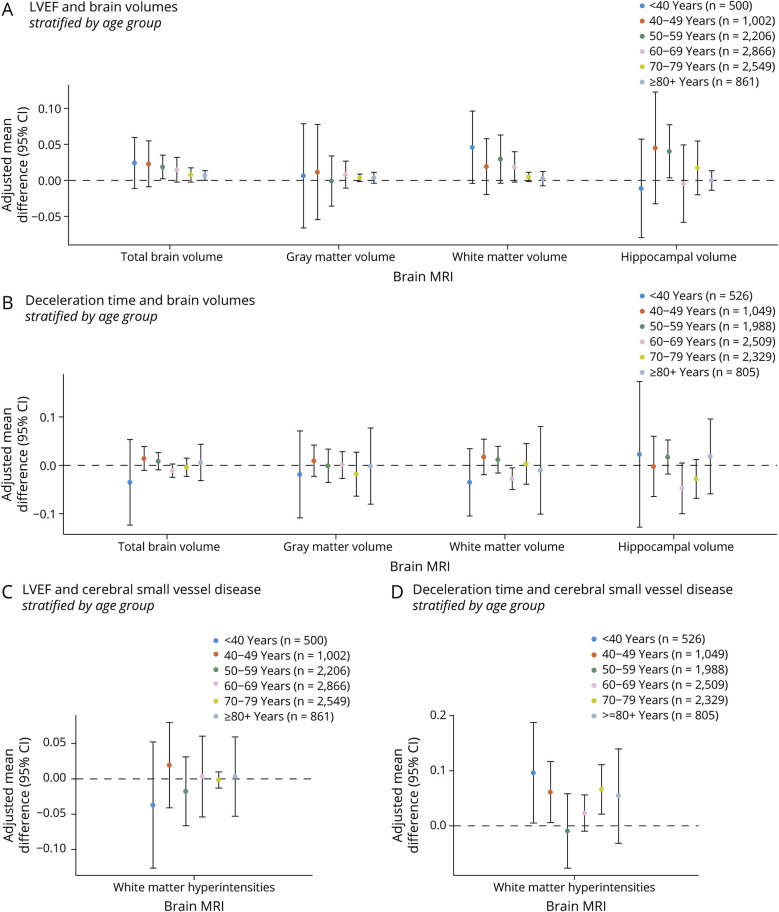
Age-Stratified Analysis of Systolic and Diastolic Function and Its Neuroimaging Correlates Age-stratified analysis of LVEF (systolic function) and deceleration time (diastolic function) with brain volumes (A, B) and cerebral small vessel disease (C, D). Results are displayed as pooled adjusted mean differences and corresponding 95% CIs (DerSimonian and Laird method) from AGES, ARIC, FHS, RS, SHIP-START, and SHIP-TREND for LVEF, and AGES, ARIC, RS, SHIP-START, and SHIP-TREND for deceleration time. The number of participants in each age category is mentioned in the legend. All echocardiographic and neuroimaging markers were standardized (displayed per 1 SD) to facilitate comparison. Model 2 is displayed, which is adjusted for age-specific, age^2^-specific, sex-specific, cohort-specific covariates, *APOE*-ε4 carriership, smoking, body mass index, ethnicity, systolic blood pressure, diastolic blood pressure, use of blood pressure–lowering medication, total cholesterol, high-density lipoprotein-cholesterol, use of lipid-lowering medication, diabetes, and history of coronary heart disease. AGES = Age, Gene/Environment Susceptibility Study; ARIC = Atherosclerosis Risk in Communities Study; CHS = Cardiovascular Health Study; FHS = Framingham Heart Study; LVEF = left ventricular ejection fraction; RS = Rotterdam Study; SHIP = Study of Health in Pomerania.

Associations between systolic or diastolic function and neuroimaging markers remained similar after excluding individuals with covert brain infarcts or clinical heart failure during sensitivity analyses. From age 40 years and older, individuals with higher LVEF (indicating better systolic function) generally showed larger brain volumes, whereas those with higher deceleration time (indicating worse diastolic function) showed more WMHs (Figure 5). Associations were less pronounced in older participants. In sex-specific analyses, women with systolic dysfunction had smaller TBVs, particularly in WM and the hippocampus, compared with men (eFigure 6).

## Discussion

In this coordinated meta-analysis of 7 community-based cohorts including 10,889 participants from midlife to late-life, we found that subclinical cardiac dysfunction and clinical heart failure were associated with brain atrophy on MRI. These associations were observed for both systolic and diastolic function, with a notable trend for HV. Diastolic dysfunction also seemed to correlate with a greater burden of WMHs on MRI, particularly when considering continuous measures like deceleration time. From midlife onward, preserved cardiac function was associated with larger brain volumes, suggesting a role in maintaining brain structure that may support cognitive health during aging.

The implications of subclinical cardiac dysfunction on brain health, in the absence of heart failure, have been poorly defined. Among other factors, this can be attributed to the heterogeneous methods applied to assess cardiac function across studies. For instance, studies that used cardiac output (heart rate x stroke volume)^28^ or cardiac index (cardiac output/body surface area)^11^ as a proxy for overall left ventricular function found that an increase of both indices was positively related to TBV. Similar results were obtained in studies that used LVEF as a measure of systolic function,^9,12,17,29^ where higher LVEF was seen with larger TBV. Although these findings are consistent with our results, many of these previous studies lacked precision in demonstrating similar associations with other structural neuroimaging markers. It is more important that neither cardiac output nor left ventricular ejection fraction is capable of quantifying the extent of diastolic dysfunction, which is present in about 50% of individuals with cardiac dysfunction.^30^

A recent population-based study has shown that application of the updated guidelines for HFpEF mainly yields identification of individuals in advanced stages of diastolic dysfunction.^20^ Results from this study suggest that even mild diastolic dysfunction is associated with adverse brain health. This is supported by the finding that a higher deceleration time was significantly associated with more WMHs, a marker of cerebral small vessel disease. Of note, the deceleration time represents the time lapse to reach equal pressure between the left atrium and the LV during diastole, where an isolated prolongation suggests impaired LV relaxation, but without an elevated filling pressure.^31^ These findings support the existence of shared mechanisms between impaired LV relaxation and vascular remodeling in the brain's small vessels, such as endothelial dysfunction, which may contribute to more WMHs.^32^

With gradual increases in LV filing pressure, especially restrictive diastolic function, that represents end-stage diastolic dysfunction with pronounced rise in LV filling pressure, we observed a significantly smaller hippocampus. Many pathophysiologic mechanisms are believed to contribute to both hippocampal atrophy and restrictive cardiomyopathy, one of them being amyloidosis.^33^ Although more than 30 different proteins may contribute to the development of amyloidosis, accumulations of amyloid fibrils β40 (Aβ-40) and Aβ-42 in the brain are regarded as classical hallmarks of dementia. Some histology reports confirmed the presence of both Aβ-40 and Aβ-42 aggregates in the heart of patients with Alzheimer disease.^34,35^ Moreover, higher levels of plasma Aβ-40 and Aβ-42 have recently been associated with burden of atherosclerosis, adverse outcomes of CHD, and risk of incident heart failure, irrespective of established cardiovascular risk factors.^36,37^ Building on previous research, which had primarily focused on the link between diastolic dysfunction and cerebral small vessel disease,^38^ these findings support the notion that there may be additional pathophysiologic mechanisms underlying the relationship between diastolic dysfunction and neurodegeneration.

The hippocampus was also significantly smaller in participants with clinically overt heart failure in our study, despite comparable atrophy in white and GM. These findings align with a previous report, where the prevalence of hippocampal atrophy among patients with clinical heart failure was as high as 34.6%.^39^ In the same study, hippocampal atrophy was found to be an independent predictor of a poor prognosis following heart failure. The latter could reflect confounding by indication because some of the patients in that study were admitted for treatment of worsening heart failure. Findings from this study suggest that hippocampal atrophy may even occur at subclinical stages of cardiac dysfunction, indicating a potential vulnerability in affected individuals. Longitudinal studies remain necessary to determine whether these structural brain changes translate into cognitive decline over time. The observed effect sizes, while modest, point to important underlying pathways that may have long-term clinical implications.

Our age-stratified analyses indicate that better systolic function in midlife, from age 40 years and onwards, is positively associated with brain volumes. Such associations were less pronounced after age 70 years. In accordance, worse diastolic function was linked to a higher burden of WMHs across all age groups. On one hand, this stresses the importance of early detection and treatment. On the other hand, the lack of associations in the older population could be due to greater heterogeneity of neurodegeneration at old age, survival or selection bias, or less precision (smaller sample sizes). Sex-specific differences between cardiac dysfunction and brain MRI measures remain poorly understood. Our findings align with previous studies suggesting systolic dysfunction may affect women more than men,^40,41^ but these potential sex or gender differences warrant further study.

Major strengths of this study include a large sample size of community-dwelling individuals, use of standardized protocols for assessing heart and brain outcomes, and availability of detailed data on potential confounders. To address the methodologic variations of previous studies, we used multiple continuous measures for assessing systolic and diastolic function. We specifically used measures that were available across all cohorts, and subsequently categorized them in a systematic manner. By including a population-based sample across the age span, we attempted to mitigate survival bias, which occurs when only individuals with clinical heart failure or the oldest-old, in whom mortality is high, are examined. Nevertheless, several limitations need to be acknowledged. First, given the cross-sectional design of this study, causal inferences and temporality of the associations cannot be established. Second, a comprehensive evaluation of diastolic dysfunction was constrained due to the absence of tissue Doppler imaging data across cohorts, or its inavailability concurrent with brain MRI. While recognizing this constraint, it is important to note that estimates derived from conventional Doppler exhibit a high correlation with measures from tissue Doppler imaging in screening for diastolic dysfunction, particularly when accounting for wall motion abnormalities, a factor that we considered (see eMethods 2). Third, differences in spatial resolution, imaging protocols, and scanner settings may have compromised the comparability of brain volume measurements. We made efforts to harmonize MRI data processing across cohorts and ensured that each cohort implemented standardized quality control procedures, yet residual variability could still influence the observed associations. Fourth, considering the many common pathways along the heart-brain axis, we cannot preclude residual confounding by factors unmeasured in this study. Fifth, the amount of participants in advanced stages of systolic or diastolic dysfunction was small. Power to detect associations in those subgroups may have been compromised, and validation in larger samples may be necessary. Yet, it is plausible that similar distributions will be found in the general population. It should also be reckoned that individuals could have both systolic and diastolic dysfunction at the same time. Sixth, we refrained from multiple testing correction in our analyses because it may reduce the chance of type I error, but at the cost of increasing the risk for type II error, especially in the presence of multiple correlated predictors (e.g., cardiac measures).^27^ The consistent results across the studies and our meta-analyses strengthened the validity of our findings. Finally, despite the fact that some cohorts included an ethnically diverse population, the majority of participants was of European ancestry, which may limit the generalizability of our findings.

In summary, subclinical cardiac dysfunction and heart failure were associated with brain imaging markers of neurodegeneration and small vessel disease in this large coordinated meta-analysis of community-dwelling individuals. With increasingly worse cardiac function, brain atrophy was similar for GM and WM, but the hippocampus seemed to be a particularly vulnerable structure. These results highlight the importance of preserving cardiac function for promoting brain health during aging and encourage further investigations into the complex interplay between cardiac and brain health.

## Glossary

AGES: Age, Gene/Environment Susceptibility Study
ARIC: Atherosclerosis Risk in Communities Study
ASPS: Austrian Stroke Prevention Study
CCC: Cross-Cohort Collaboration
CHD: coronary heart disease
CHS: Cardiovascular Health Study
DT: deceleration time
EA-ratio: peak blood flow velocity of early diastole (E-top) / late diastole (A-top)
FHS: Framingham Heart Study
FS: fractional shortening
GM: gray matter
HDL: high-density lipoprotein
HFpEF: heart failure with preserved ejection fraction
HV: hippocampal volume
LV: left ventricle
LVEDD: left ventricular end-diastolic diameter
LVEF: left ventricular ejection fraction
RS: Rotterdam Study
SHIP: Study of Health in Pomerania
TBV: total brain volume
WM: white matter volume
WMH: white matter hyperintensity
